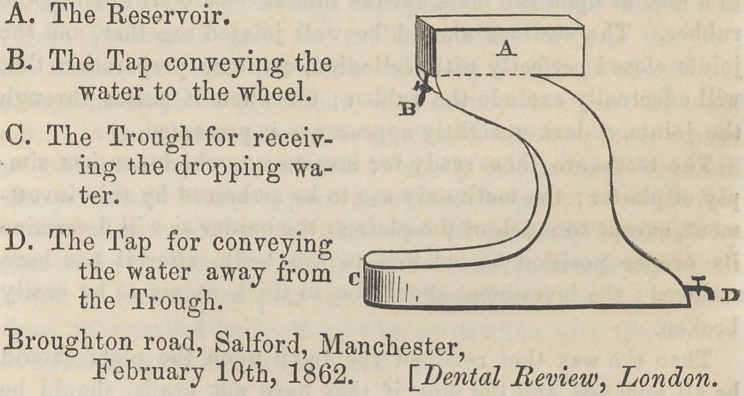# Improved Water Apparatus for Lathe

**Published:** 1862-05

**Authors:** Wm. Hopkinson


					﻿Improved Water Apparatus For Lathe—By Wm. Hopkinson, M.C.D.E.—I forward a description of a new Water
Apparatus, with trough, for the Lathe. Having never seen
any thing like it before, and finding it both clean and useful,
I think the profession may adopt it with confidence as an im-
provement upon all other Water Apparatus in use. It is con-
structed of thin sheet brass, or copper, and any copper-smith
or tinman can make one. The apparatus has a reservoir with
a stop-tap for dropping the water on the emery wheel; while
the trough below has a stop-tap behind for letting out the
water, which runs into it from above. I send you a sketch
of the apparatus, so that it may convey the idea to your read-
ers. Its higlit, from the bottom of the trough to the top of
the reservoir, is nine inches and a quarter; the trough is three
inches wide, one inch and three- quarters deep, and four inches
in length; while the outer side or flange is eight inches long,
so as to steady it firm in the bench. The reservoir above is
three inches broad, two inches deep, and four inches long ;
having in front, and looking downwards, a stop-tap for con-
veying the water to the wheel on the Lathe ; of course the
hight or width may be altered to suit the headstock of the
Lathe.
We have used for several years a reservoir similar in some
respects to the above, and found it more convenient for the
purpose than any thing we have ever used. We think the
form here given is rather better than our own, and intend to
have one constructed.—Ed. Reg.
				

## Figures and Tables

**Figure f1:**